# Complex folding and misfolding effects of deer-specific amino acid substitutions in the β2-α2 loop of murine prion protein

**DOI:** 10.1038/srep15528

**Published:** 2015-10-22

**Authors:** Sonya Agarwal, Kristina Döring, Leszek A. Gierusz, Pooja Iyer, Fiona M. Lane, James F. Graham, Wilfred Goldmann, Teresa J. T. Pinheiro, Andrew C. Gill

**Affiliations:** 1 The Roslin Institute and R(D)SVS, University of Edinburgh, Easter Bush Veterinary Centre, Roslin, Edinburgh EH25 9RG, UK; 2 School of Life Sciences, University of Warwick, Gibbet Hill Road, Coventry, CV4 7AL, UK

## Abstract

The β2–α2 loop of PrP^C^ is a key modulator of disease-associated prion protein misfolding. Amino acids that differentiate mouse (Ser169, Asn173) and deer (Asn169, Thr173) PrP^C^ appear to confer dramatically different structural properties in this region and it has been suggested that amino acid sequences associated with structural rigidity of the loop also confer susceptibility to prion disease. Using mouse recombinant PrP, we show that mutating residue 173 from Asn to Thr alters protein stability and misfolding only subtly, whilst changing Ser to Asn at codon 169 causes instability in the protein, promotes oligomer formation and dramatically potentiates fibril formation. The doubly mutated protein exhibits more complex folding and misfolding behaviour than either single mutant, suggestive of differential effects of the β2–α2 loop sequence on both protein stability and on specific misfolding pathways. Molecular dynamics simulation of protein structure suggests a key role for the solvent accessibility of Tyr168 in promoting molecular interactions that may lead to prion protein misfolding. Thus, we conclude that ‘rigidity’ in the β2–α2 loop region of the normal conformer of PrP has less effect on misfolding than other sequence-related effects in this region.

Transmissible spongiform encephalopathies (TSEs), also known as prion diseases, are neurodegenerative diseases that include Creutzfeldt-Jakob disease, scrapie, bovine spongiform encephalopathy and chronic wasting disease (CWD). TSE disease is characterised by the formation of proteinaceous deposits composed of a misfolded form (PrP^Sc^) of the cellular prion protein, PrP^C^. It is widely believed that PrP^Sc^ carries with it enough information encoded in its structure to initiate disease in naïve individuals after experimental inoculation^1^,^2^. The prion protein is the product of the *PRNP* gene and comprises roughly 250 amino acids, depending on species. The mature protein carries a C-terminal glycosylphosphatidylinositol membrane anchor, two N-linked carbohydrate chains and a single disulphide bond. The NMR structure of PrP^C^ shows that the mature N-terminal domain (residues 23 to ~120) of the protein is flexibly-disordered and contains poly-L-proline conformational elements^3^,^4^, whilst the C-terminal region (residues ~121 to 231) consists of 3 α-helices and two short β-strands^5^,^6^. Figure 1 displays many of these structural features schematically. When PrP^C^ misfolds into PrP^Sc^, there is a dramatic increase in β-sheet structure at the expense of helical structure^7^ and the resultant protein becomes partially resistant to proteolytic digestion. Crucially, post-translational modifications appear similar between PrP^C^ and PrP^Sc^, suggesting that the conversion process is purely conformational^2^,^8^.

Alterations to specific amino acids of PrP^C^, both within and between species, can impact on the course of TSE disease. For instance, differences in specific residues between species are believed partly to underlie species barrier effects in disease transmission^9^,^10^, whilst coding polymorphisms in the *PRNP* gene in humans and animals have been linked to susceptibility to acquired TSE infections^11^,^12^. Many studies have sought to define differences in structural properties of recombinant PrP (recPrP) carrying amino acid changes relative to the wildtype counterparts, but there exists poor overall consensus on how specific amino acid changes affect mechanisms of protein misfolding. In recent years, attention has focussed on a key structural loop of PrP^C^ located between β-sheet 2 and α-helix 2 (see Fig. 1)^13^,^14^,^15^,^16^,^17^,^18^,^19^,^20^,^21^,^22^. This interest was initiated following the publication of NMR data suggesting that the β2–α2 loop of cervid PrP^C^ was particularly well defined relative to that of other mammalian species. Cervid and other prion proteins, for which the loop structure could be defined, were thus given the designation ‘rigid loop’ PrP^23^ and it was suggested that the structures of non-rigid β2–α2 loops could not be defined because rapid conformational exchange caused NMR peak broadening. Gossert *et al.* confirmed that the ‘rigid loop’ phenotype of the β2–α2 loop in cervid PrP^C^ was caused by residues 169 and 173 (murine numbering, which will be used from this point forward)^23^. However, more recent data has suggested that ‘rigid loop’ prion proteins are actually undergoing extremely rapid conformational interconversion between two discrete states, such that an average structure is being measured by NMR^24^. The implication is thus that ‘rigid loop’ PrP molecules are actually more conformationally labile than non-‘rigid loop’ proteins, but the definition of a ‘rigid loop’ still differentiates prion proteins whose loop conformation is resolved by NMR at 20 °C from those that aren’t.

Chronic wasting disease is a TSE of deer and elk that shows high natural transmissibility^25^. Since cervid PrP^C^ possesses ‘rigid loop’ properties, it has been suggested that a rigid β2-α2 loop may explain the high susceptibility of cervids to CWD^14^. To investigate mechanistically whether a ‘rigid loop’ causes biophysical changes in PrP that may impact on disease, we have investigated the stability and misfolding pathways of wildtype murine recPrP (recMoPrP), which is not a ‘rigid loop’ structure, as well as a murine recPrP containing the deer-specific amino acids at codons 169 and 173 (recMoPrP-S169N/N173T), which has been shown to exhibit ‘rigid loop’ behaviour^23^. Two additional recombinant proteins were produced in which single amino acid changes were made (recMoPrP-S169N, a ‘rigid loop’ molecule^26^, and recMoPrP-N173T, a non-‘rigid loop’ molecule^23^). The data produced do not support a role for loop rigidity in mediating misfolding *per se,* but instead indicate a complex interplay between stability and the different misfolding pathways underpinned by variable solvent exposure of Tyr168.

## Results

**Figure 1:**
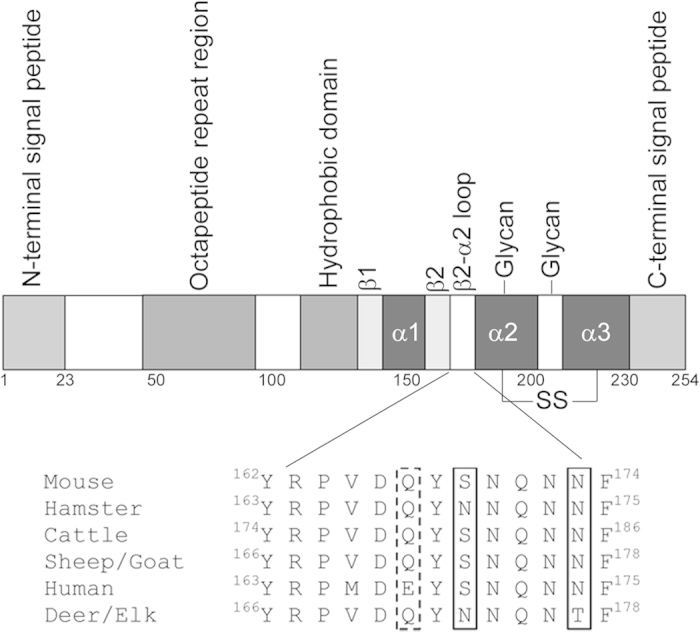
Schematic diagram of structural features of mammalian prion proteins (top). Sequence numbering is per the murine protein. Also depicted is an alignment of the sequences of important mammalian prion proteins in the β2–α2 loop region—sequence numbering on this part of the figure is precise and is taken from the protein sequences of each individual species. Boxes with solid lines represent the key amino acid differences between deer/elk and other species whilst the dashed box represents the polymorphic position in sheep that segregates with susceptibility to prion disease infection.

We have previously demonstrated that combined *in vitro* and *in silico* studies can be used to understand the structural and functional effects of amino acid changes at codon 164 in murine PrP^27^,^28^. Residue 164 is adjacent to the β2–α2 loop region (Fig. 1) and in the current study we investigate cervid-specific amino acid substitutions in this region, specifically at residues 169 and 173. The 4 protein variants were expressed, purified and the integrity of each was checked routinely by SDS-PAGE, mass spectrometry and circular dichroism spectropolarimetry. There were minor differences in the far-UV CD spectra of the four protein variants (Supplementary Fig. 1), which may suggest minor variations in the amount of tertiary structure present, but qualitatively all show the characteristic double minima at 222 and 208 nm indicative of protein with a high level of helical structure. We therefore used our proteins to investigate the differences in protein stability and misfolding caused by the cervid mutations by use of a range of biochemical and biophysical techniques, thereby allowing us to truly assess whether β2–α2 loop conformation dictates protein stability.

### The S169N substitution dramatically affects protein fibrillisation, but its effect is potentiated by co-inclusion of the N173T substitution

**Figure 2:**
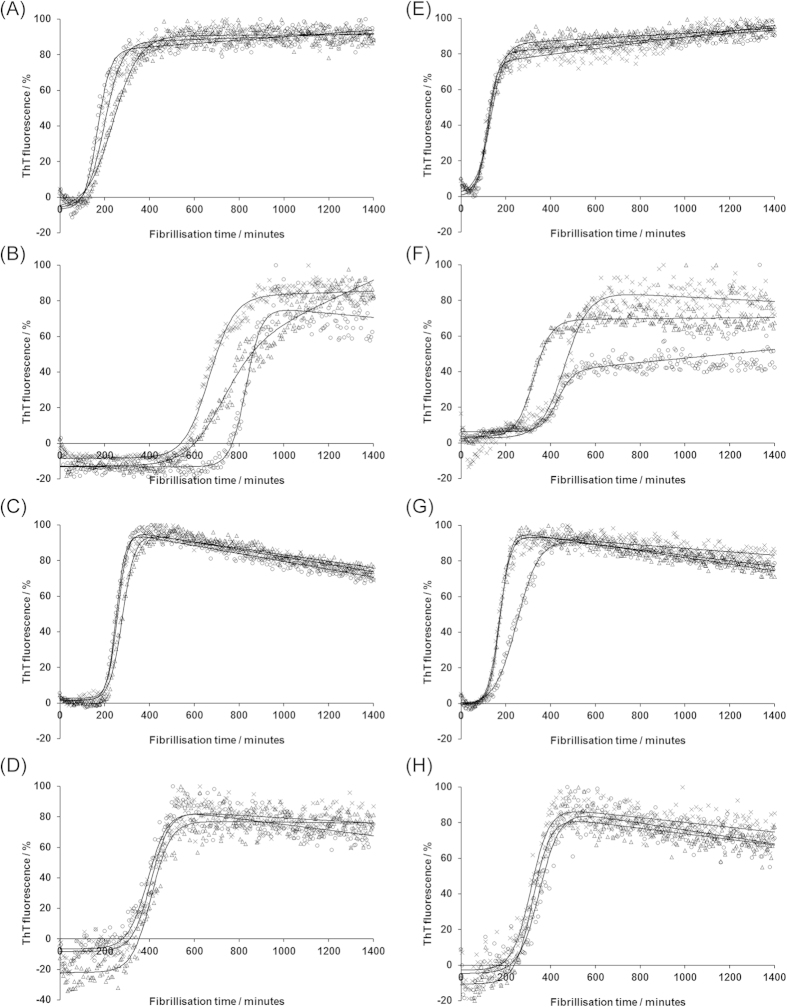
Representative profiles of ThT fluorescence as a function of fibrillisation time for reactions involving (A) and (E) wildtype recMoPrP; (B) and (F) recMoPrP-S169N; (C) and (G) recMoPrP-N173T; (D) and (H) recMoPrP-S169N/N173T. Reactions depicted in panels (**E–H**) included the addition of preformed fibrils at time zero, whilst those in panels (**A–D**) were unseeded. Triplicate fibrillisation reactions were monitored for 24 hours with fluorescence readings taken every 5 minutes and plotted as points (Δ,ο,x) to which sigmoidal curves were fitted (solid lines).

**Figure 3:**
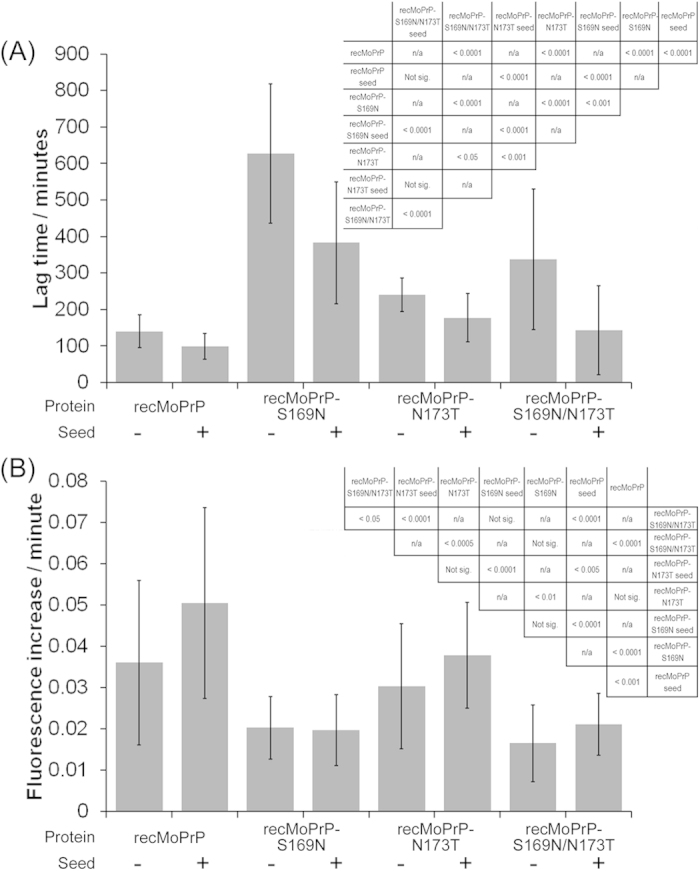
Graphs of quantitative parameters extracted from fitted fibrillisation curves for the 4 protein variants both without and with addition of preformed seed to drive the reaction. (**A**) is a bar chart depicting the average lag time to fibrillisation whilst (**B**) represents the average rate of fibrillisation. Error bars are standard deviations and represent n > = 18 reactions for each variant. T-Test statistics (inset tables) test differences between lag times (**A**) or elongation rates (**B**).

Firstly we investigated the effect that the amino acid changes had on fibrillisation of the proteins (Figs 2 and 3). Fibrillisation was monitored by ThT fluorescence and, to allow comparisons between proteins, background fluorescence was removed and the resulting signals normalised. In our buffer system, fibrillisation is characterised by a lag phase, during which a nucleus of misfolded protein forms, followed by an exponential elongation phase until most protein is contained within fibrils and the trace plateaus. Addition of a pre-formed seed typically shortens the lag phase by by-passing the nucleation event. Representative fibrillisation traces are given in Fig. 2 whilst bar charts depicting average lag times and elongation rates, for at least 18 experimental repeats, are shown in Fig. 3. Figure 3 also incorporates relevant student’s t-tests. For all protein variants, we demonstrated that increases in ThT fluorescence indicated fibril formation by confirming the presence of a 17 kDa proteinase K resistant species following maturation (Supplementary Fig. 2), a property that is indicative of *bona fide* fibrils formed from recombinant PrP^29^.

RecMoPrP fibrillised rapidly with a lag time of ~2 hours (Fig. 3(A)), as published previously^30^, which was shortened slightly by adding pre-formed fibrils to seed the reaction. By contrast, recMoPrP-S169N had an extended lag phase to fibrillisation, which was also reduced by addition of pre-formed seed, although the lag phase of seeded reactions of recMoPrP-S169N were still substantially longer than those of recMoPrP. Lag phases for recMoPrP-N173T, both with and without seed, were also increased relative to recMoPrP, but the differences were small, whilst recMoPrP-S169N/N-173T had lag phases that were intermediate between the two singly-mutated proteins.

Considering the elongation phase of fibrillisation, recMoPrP had the highest elongation rates, and those of recMoPrP-N173T were similar. By contrast, both recMoPrP-Sn169 and recMoPrP-S169N/N173T had elongation rates that were reduced relative to recMoPrP and recMoPrP-N173T. Thus, overall, the N173T substitution does not dramatically affect fibrillisation whilst the S169N substitution slows both nucleation and elongation. When both mutations are present in the same protein, fibrillisation properties are intermediate between the two single mutants. From these studies it appears that the two proteins that have the ‘rigid loop’ phenotype are poor substrates for misfolding but we wanted to confirm this conclusion using an alternate misfolding assay.

### The doubly mutated protein, recMoPrP-S169N/N173T, oligomerises more rapidly and produces more high molecular weight oligomers than other proteins

**Figure 4:**
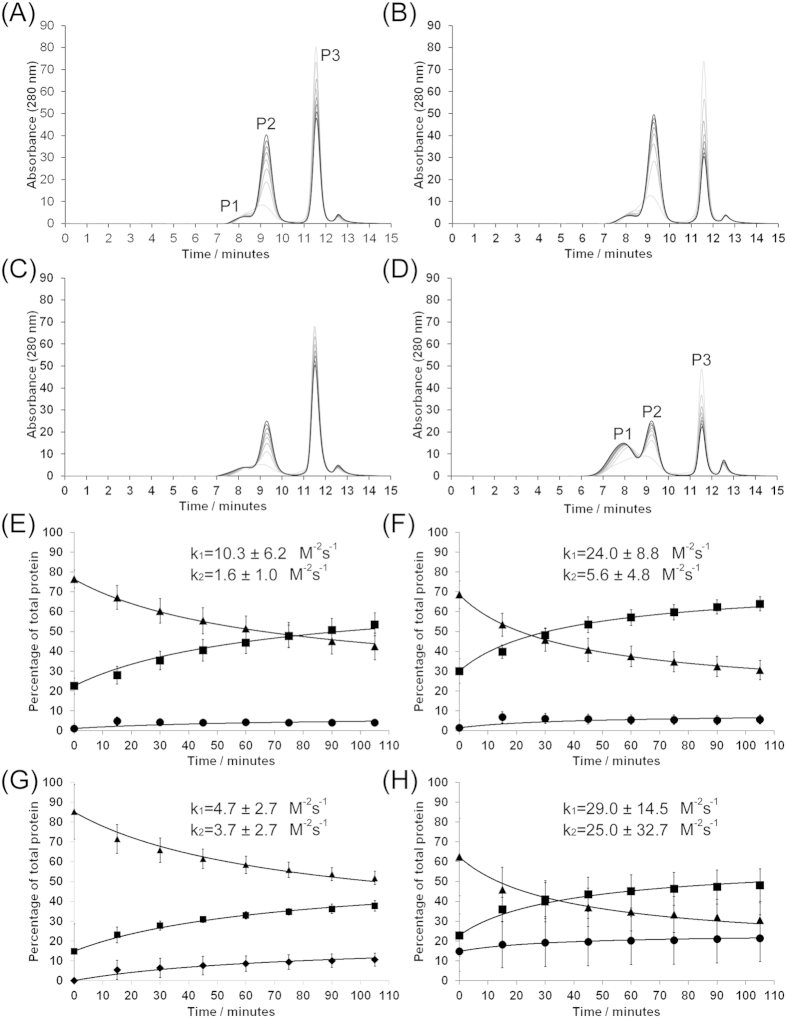
Oligomerisation of the murine PrP variants. Panels (**A–D**) represent typical size exclusion chromatograms over a time course of oligomerisation of (**A**) recMoPrP; (**B**) recMoPrP-S169N; (**C**) recMoPrP-N173T; (**D**) recMoPrP-S169N/N173T. Samples were incubated at 45 °C in oligomerisation buffer and aliquots were removed from the reaction every 15 minutes from 0 (lightest trace) to 105 minutes (darkest trace) for analysis. Oligomerisation was performed at least 4 times for each variant, the area under each peak was calculated and plotted as a function of time for (**E**) recMoPrP; (**F**) recMoPrP-S169N; (**G**) recMoPrP-N173T; (**H**) recMoPrP-S169N/N173T. •–peak 1 (large oligomer) ■–peak 2 (small oligomer) ▲–peak 3 (monomeric protein). Error bars represent the standard deviations.

We next investigated a misfolding pathway that results in comparatively low molecular weight, oligomeric species and that is driven primarily by reduced pH^27^,^31^. The extent of oligomerisation was monitored by size exclusion chromatography and typical sets of data for each of the 4 protein variants are shown in Fig. 4 (A–D). For recMoPrP (Fig. 4(A)), the peak corresponding to monomeric protein, eluting at 11.5 minutes (labelled P3), gradually reduced in intensity over the reaction, whilst a new peak eluting at ~9 minutes (labelled P2) increased in intensity. Previously, this peak has been shown to correspond to oligomers of roughly 300 kDa (around 12-meric), whilst a shoulder on this peak at 8 minutes (labelled P1) represents a still higher molecular weight form composed of ~36 monomer units^31^. The peak at 12.5 mins corresponds to the total volume of the column and is caused by variations in salt concentration.

Oligomerisation profiles of each of the proteins show similar features but quantitative differences, hence we repeated the analyses at least 6 times for each proteins and calculated the average percentage of each molecular species. This allowed curves to be fitted to the data according the reaction scheme given in Supplementary Materials (Fig. 4 (E–H)). It should be born in mind that there was a delay of up to 10 minutes between preparing the oligomerisation reaction and performing the first SEC analysis, hence the x-axes in these charts are actually offset compared to the time after sample preparation. This offset explains why “time zero” samples already contain significant amounts of oligomers and why the prevalence of oligomers in the starting samples correlates with the speed of oligomerisation. The fitting procedure allowed for variations in the time it took to prepare samples and this allowed us to extract rate constants for the reactions that yield the small (k_1_) or large (k_2_) oligomers, which are displayed on Fig. 4. An associated table (Supplementary Table 1) contains details of where differences in rate constants are statistically significant.

Oligomerisation profiles for recMoPrP-N173T (Fig. 4(C)) are similar to those for recMoPrP, and the rates of oligomer formation for these proteins are not statistically different. recMoPrP-S169N oligomerises more rapidly than recMoPrP and differences in k_1_ are statistically significant (Supplementary Table 1). In contrast to the other 3 proteins, recMoPrP-S169N/N173T (Fig. 4(D)) lost monomer from the oligomerisation reaction rapidly and increasing amounts of the larger oligomer, P1, eluting at 8 minutes, were produced at the expense of the smaller oligomer, P2. However, oligomerisation profiles were rather variable and, whilst differences in k1 relative to recMoPrP are statistically significant, those in k2 are not. Overall, the recMoPrP-S169N and recMoPrP-S169N/N173T protein oligomerized significantly more rapidly than the wildtype recMoPrP (differences in k_1_ are significant, p < 0.05) suggesting that a ‘rigid loop’ phenotype predisposes the protein to misfolding. This conclusion is the opposite to that obtained for fibrillisation but actually concurs with similar observations that we made for proteins that varied at codon 164^27^.

### The S169N substitution reduces thermal stability, but its effect is reversed by concomitant N173T substitution

**Figure 5:**
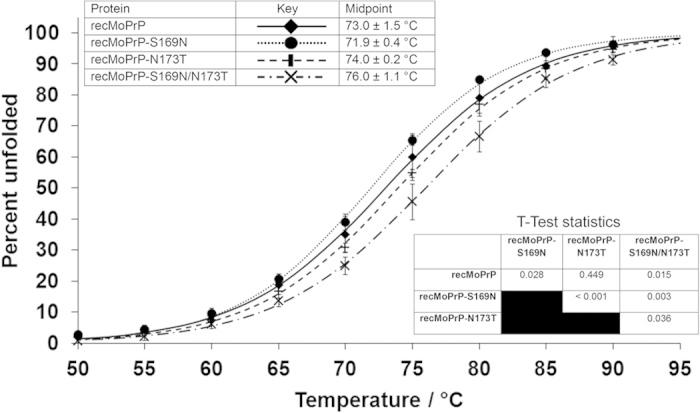
Thermal denaturation profiles of recombinant PrP variants as monitored by circular dichroism spectropolarimetry at 222 nm. The normalised ellipticity at 222 nm is plotted against temperature and a sigmoidal curve fitted. Error bars are standard deviations from n > = 5 replicate analyses. T-Test statistics test differences between midpoints of each curve.

For recPrP mutants that differed at amino acid 164, we previously identified a correlation between resistance to thermal denaturation and the ability of protein variants to convert into disease-relevant misfolded isoforms^27^. *In vitro* oligomerisation proceeded more rapidly for thermally stable proteins, whilst *in vitro* fibrillisation was less rapid for thermally-stable proteins^28^. To determine whether differential thermal stability could contribute to the alteration in fibrillisation and oligomerisation rates between the current protein variants, we used circular dichroism to determine how the proportion of protein that was unfolded varied with temperature. At least 4 experimental replicates were done for each variant and the average of these data is shown in Fig. 5. The significance of differences in the midpoints of the thermal transitions was tested using Student’s t-test.

Similar to the oligomerisation data, the thermal denaturation profiles for the doubly-mutated protein, recMoPrP-S169N/N173T were more variable than those for the other protein variants, but nevertheless, this protein was significantly more thermally stable than either recMoPrP or recMoPrP-S169N. The midpoint of the thermal transition of recMoPrP-N173T was not significantly different from recMoPrP, whilst recMoPrP-S169N was significantly less stable than the wildtype protein. Therefore, in line with our previous studies, an increase in thermal stability of recMoPrP-S169N/N173T relative to recMoPrP correlates with a reduction in fibrillisation kinetics but an increase in oligomerisation kinetics, whilst there are no significant differences in thermal stability of recMoPrP-N173T and recMoPrP and their oligomerisation and fibrillisation profiles were also similar. However, the thermal destabilisation of recMoPrP-S169N relative to recMoPrP was unexpected in light of the fibrillisation and oligomerisation data. We therefore sought an alternative assay to assess the relative stability of the different protein variants.

### RecMoPrP-S169N and recMoPrP-S169N/N173T are more susceptible to urea-based denaturation than recMoPrP or recMoPrP-N173T

**Figure 6:**
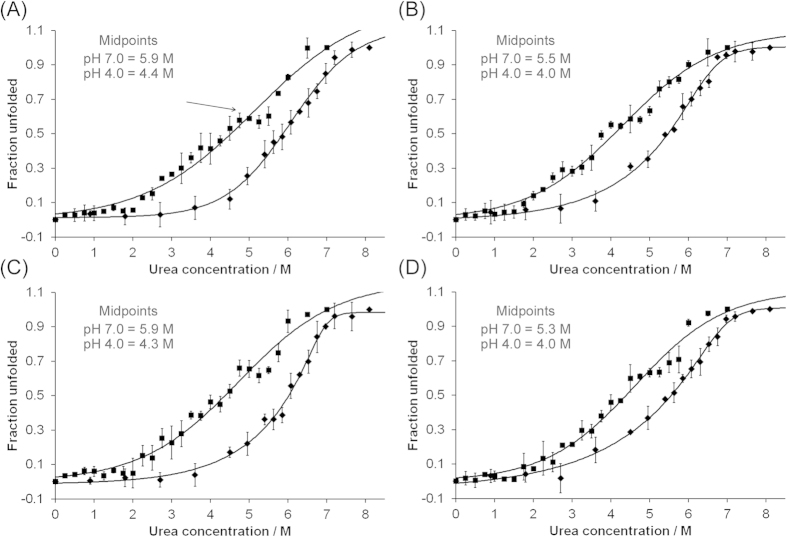
The unfolding transition of PrP variants in the presence of urea at pH 7 (♦) and pH 4 (■) at a concentration of 5 μM. The ellipticity at 222 nm was monitored by circular dichroism, normalised, and the average of 3 readings were plotted against the increasing concentration of urea. A curve representing a bi-phasic transition was fitted to each set of data to allow the calculation of midpoints of the transitions (**A**) recMoPrP; (**B**) recMoPrP-S169N; (**C**) recMoPrP-N173T; (**D**) recMoPrP-S169N/N173T.

Monitoring the circular dichroism spectrum of recPrP as a function of urea concentration has previously allowed the identification of β-structured intermediates in the unfolding pathway^32^. We used urea-based denaturation to study the unfolding transition of our protein variants and the data are plotted graphically in Fig. 6. Each data point is the average of three experimental replicates but a single sigmoidal curve has been fitted through the average of the data for each protein variant, assuming a two state unfolding reaction. Although this process allowed the extrapolation of the midpoint of the unfolding transition in each case, the fit to the data was poor, since there are intermediates in the unfolding transition, hence we restrict ourselves to a qualitative interpretation of these data.

Urea-mediated unfolding was studied at two different pH values and these were chosen such that the higher pH was close to that used during thermal denaturation and fibrillisation studies, whereas the lower pH was close to that used during the oligomerisation experiments. Since unfolding intermediates had previously been identified at reduced pH using similar methodology, we included additional data points at pH 4.0 to allow us to discern such intermediates, if they were present. At both pH 4.0 and 7.0, all recPrP proteins produced urea denaturation profiles which were essentially sigmoidal, as shown in Fig. 6, but which differed in terms of the midpoints of the unfolding transitions. At pH 7, recMoPrP and recMoPrP-N173T produced denaturation curves that were centred on ~5.9 M urea. By contrast, the unfolding of recMoPrP-S169N and recMoPrP-S169N/N173T had midpoints at ~5.5 M and ~5.3 M, respectively. At pH 4.0, midpoints of the transitions for recMoPrP and recMoPrP-N173T were again similar (~4.4 M and ~4.3 M respectively), whilst the midpoints of the unfolding transitions for recMoPrP-S169N and recMoPrP-S169N/N173T were reduced to ~4.0 M urea.

The data at pH 4, depicted in Fig. 6, also indicate the presence of unfolded intermediates for all proteins studied, as evidenced by plateaus in the unfolding transitions (arrowed in Fig. 6(A)). Although several such plateaus may exist, the most obvious plateau for recMoPrP, recMoPrP-N173T and recMoPrP-S169N/N173T occurred at ~5 M urea, whilst for recMoPrP-S169N this plateau occurred at ~4.5 M urea. Thus, taken together, these data indicate that both recMoPrP-S169N and recMoPrP-S169N/N173T are more susceptible to urea-based denaturation than recMoPrP and recMoPrP-N173T, whilst all 4 protein variants form unfolding intermediates at pH 4.0.

In all our *in vitro* experiments, recMoPrP-N173T behaved similarly to recMoPrP, whilst results for the other two proteins depend on the unfolding/misfolding pathway being studied. Coupled with our previous results^27^, the current results suggest that the different properties of recMoPrP-S169N and recMoPrP-S169N/N173T are caused partly by structural stability of the normal form of the protein. However, the actual amino acid sequence (as opposed to structure of the β2–α2 loop) clearly plays some role, probably by determining the detailed intra- or inter-molecular interactions during different unfolding or misfolding pathways.

### Molecular dynamics simulations confirm low stability of MoPrP-S169N relative to other PrP variants

**Figure 7:**
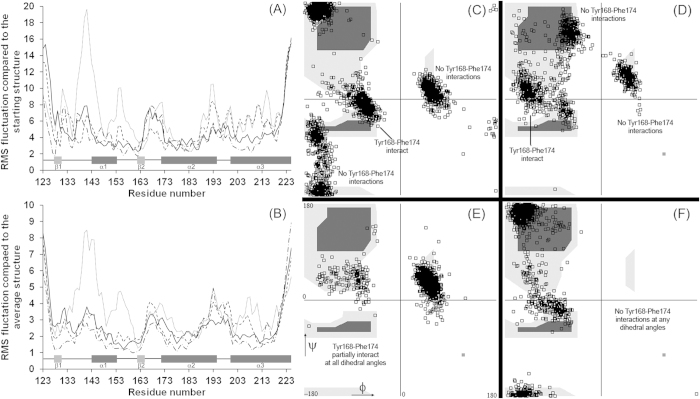
Root mean square deviations of the protein backbone grouped according to amino acid relative to (A) the starting structure (wild type murine protein pdb code 1AG2) and (B) compared to the average structure calculated for each protein. Solid line represents data from 2 replicate runs of the wildtype MoPrP; dotted line is MoPrP-S169N; dashed line is MoPrP-N173T; dot-dash line is MoPrP-S169N/N173T. Ramachandran plots of the φ–ψ dihedral angles of the backbone of the Ser/Asn169 amino acid for structures output over the course of molecular dynamics simulations of (**C**) MoPrP—structures output from both the previous and new simulations are used (**D**) MoPrP-S169N (**E**) MoPrP-N173T (**F**) MoPrP-S169N/N173T.

To understand, at an atomic level, the effect that each amino acid substitution has on the structure and stability of the normal form of PrP, we undertook molecular dynamics simulations on the wildtype protein (MoPrP) as well as proteins that we mutated *in silico* to include the substitutions S169N, N173T or both. Root mean square deviation of α-carbon atoms from either the starting structures or the average structure were calculated for each simulation and the results are shown in Fig. 7, panels (A) and (B). MoPrP, MoPrP-N173T and MoPrP-S169N/N173T show relatively small deviations from either the starting structure or average structure in many regions of the protein, although the loop regions joining areas of globular structure show the most dynamic excursions. By contrast, MoPrP-S169N has significant deviations from both its starting structure and average structure in several regions of the protein, most noticeably residues 134–164 and helix 3 (residues 204–214). This is suggestive of a decrease in stability of specific helical sections caused by the S169N mutation and such an effect was also observed during determination of the NMR structure of this protein^26^. These findings provide a structural rationale for the reduced thermal stability of recMoPrP-S169N relative to the other proteins studied.

### Structural polymorphism in the β2-α2 loop backbone suggests that Tyr168 bonding may differentiate PrP isoforms

We next investigated the predicted structures of the β2–α2 loop regions of each of the proteins. Initially, we calculated the average structures over the course of the MD simulations and noticed that the average structure of MoPrP differed somewhat from our previously published work^27^. Averaged structures from the four proteins studied here, alongside our previously-determined structure of MoPrP, are presented schematically in Supplementary Fig. 3. In all cases, the side chains of Arg163 and Phe174 occupy the intra-loop space, but other side chains also occupy this space to rather different extents for different proteins. For instance, in the average structures of the wildtype protein MoPrP and that of MoPrP-S169N/N173T, Asn170 protrudes from the loop and is predominately solvent exposed whereas in MoPrP-N173T, Asn170 is more hidden and is located near to Arg163.

We analysed the entire trajectory for each simulation to determine whether differences in average structures arose because proteins were stable and different across each simulation or because the structures alternated between one of several conformations but spent different lengths of time (on average) in one conformation relative to another. We calculated the dihedral angles of residues of the loop region using 1000 sets of coordinates that were output every 10 ps (for the wildtype simulations we combined structures from the analysis performed in this work with structures generated in previous work) and results are presented as Ramachandran plots in Supplementary Fig. 4. φ–ψ angles for residues 165 and 166 are clustered around the archetypal “helical” area of the plot for all proteins, in line with NMR structures. However, φ–ψ angles for residues 165 and 167 occur in a bi-phasic distribution for MoPrP, MoPrP-S169N and MoPrP-N173T and these are consistent with residues 165–167 forming either a 3–10 helix or a β-turn, as has previously been suggested^24^. The double mutant, MoPrP-S169N/N173T shows φ–ψ angles for residue 167 that correspond only to β-turn.

In contrast to residues 165–167, residues 169 and 170 (and to a lesser extent residue 168) sample a wider distribution of dihedral angles in each of the proteins. Ramachandran plots for these residues have several focal areas in which φ–ψ angles are concentrated, indicating that the backbone in this region is not inherently ‘flexible’ but can adopt more than one discrete conformation. The Ramachandran plots for residue Ser/Asn169 for each of the four protein variants are shown in Fig. 7 (C–F) and illustrate the polymorphic nature of the backbone in this loop region. φ–ψ angles for residue Ser169 of MoPrP are located around the helical area (φ ~ −60, ψ ~ −20), around the β-structure area (−150, 150) but also in the left-handed helix area (60, 20). Residue 169 of the other protein variants also occupy similar areas of the plot, although for MoPrP-S169N the polyproline area (−60, 120) is occupied at the expense of the β-structure area and the double mutant protein, MoPrP-S169N/N173T, does not occupy the left-handed helix area. Overall, these data indicate that residues 168–170 of each protein can adopt specific structures that are either elongated or partially helical and that the ensembles of different structures can be distinguished by the φ–ψ angles of residue 169, depicted on Fig. 7(C–F).

When one analyses the interactions of the amino acids in the loop region as a function of the φ–ψ angles of residue 169 there are only minor differences in the detailed interactions. However, a clear difference that stands out between protein variants is the proximity of Tyr168 to Phe174. In general, when residues 168–170 are in a turn-like conformation (residue 169 φ–ψ angles are −80, 0) then the side chain of Tyr168 interacts with Phe174, through hydrophobic ring stacking, and the tyrosine hydroxyl interacts with other polar side chains of the loop region. On the other hand, when residues 168–170 adopt a more elongated conformation (residue 169 φ–ψ angles in the β-structure region or α_L_ region) the side chain of Tyr168 protrudes from the loop and is significantly more exposed to solvent. Interestingly, regardless of the φ–ψ angles of residue 169, in all structures of MoPrP-S169N/N173T Tyr168 does not interact with Phe174 and is excluded from the intra-loop region, probably as a direct result of interactions between the side chains of Asn169 and Thr173. Supplementary Fig. 5 shows the distance between the Cζ atoms of Tyr168 and Phe174 as a function of the phi-psi angles for each of the protein structural ensembles, illustrating the dependence of backbone conformation on this interaction for MoPrP and MoPrP-S169N. For MoPrP-N173T, which segregates into two distinct conformations around residue 169, interactions between Tyr168 and Phe174 appear to occur irrespective of residue 169 dihedral angles, suggesting that other bonding interactions in the intra-loop region are also stabilising the loop in this protein. Nevertheless, our data support a role for Tyr168-Phe174 ring stacking and exposure of the Tyr168 sidechain in mediating differential unfolding of PrP under particular denaturing conditions.

## Discussion

There is a significant body of evidence that suggests that the β2–α2 loop plays a key role in the disease-associated misfolding of PrP during prion diseases^21^,^33^,^34^. Transgenic mice expressing murine PrP^C^ with the substitutions S169N and N173T developed a spontaneous neurological disorder resembling TSE disease^35^, and these substitutions also affected cross-species disease transmission^15^. The S169N/N173T substitutions were chosen because they produce a ‘rigid loop’ phenotype in PrP^C^, such that the loop region is structurally well defined in NMR studies (at 20 °C)^23^, whilst in non-‘rigid loop’ prion proteins, including the murine wildtype sequence, the loop conformation is ill-defined as a result of line-broadening during NMR measurements. Furthermore, a second line of transgenic mice overexpressing murine PrP with the D166S mutation, which causes the β2–α2 loop also to be well-defined, developed spontaneous disease^16^. Thus, it has been suggested that ‘rigid loop’ prion proteins may undergo misfolding more readily *in vivo* and that animals possessing ‘rigid loop’ PrP^C^ may be more susceptible to TSE disease as a result. Deer, elk and bank voles are examples of animals possessing ‘rigid loop’ prion proteins that appear highly susceptible to disease; CWD infects cervids readily and bank voles have exceptionally short incubation periods to experimental prion infection. However, pigs, rabbits and horses also have ‘rigid loop’ prion proteins^36^,^37^,^38^ and these animals are believed to be rather resistant to TSE infections. In addition, transgenic mice expressing the entire elk prion sequence do not get spontaneous prion disease^39^,^40^ nor are they generally highly-susceptible to a range of TSE infections^41^,^42^. It remains uncertain, therefore, whether the ‘rigid loop’ phenotype is really the cause of increased susceptibility to TSE disease, or whether it is the specific amino acid alterations used to create ‘rigid loop’ PrP^C^ that impact on the misfolding process. This controversy is compounded by recent suggestions that the previous interpretation of structural rigidity in the β2–α2 loop region is incorrect. The same authors who originally proposed this theory found that the presence of resonances from the β2–α2 loop is inversely temperature-dependent^24^. This suggests that prion proteins whose NMR spectra include resonances from the β2–α2 loop region may undergo extremely rapid inter-conversion between two discrete structures, whereas for those proteins that show peak broadening in resonances from this region the conformational exchange is slower. Hence, the ‘rigid loop’ PrPs may actually be conformationally more flexible than other PrP molecules, at least within the specific region of the β2–α2 loop^24^.

Regardless of whether the ‘rigid loop’ phenotype is caused by increased structural rigidity or increased structural flexibility, the question of whether the ‘rigid loop’ phenotype causes misfolding in recombinant prion protein remains valid. The 4 proteins studied in this work produce different properties and our findings are summarised in Table 1. Compared to the wildtype recMoPrP, the ‘rigid loop’ prion protein recMoPrP-S169N/N173T was more stable to thermal denaturation, less stable to urea-induced denaturation, oligomerised more readily but fibrillised less readily. Increased levels of oligomer formation are consistent with an increased prevalence of misfolding caused by the ‘rigid loop’ and also agree with similar published studies^18^,^19^,^20^, whilst the finding that fibrillisation was inhibited further suggests that this specific misfolding pathway is of only tangential relevance to TSE disease^28^. Overall for recMoPrP-S169N/N173T, our findings conform to our previous suggestions that proteins that are more stable to thermal denaturation also undergo disease-specific misfolding more readily^27^.

When we analysed the singly-mutated protein recMoPrP-N173T we found little difference in unfolding or misfolding properties compared to the wildtype structure, suggesting that this mutation in isolation is neutral in terms of protein structure. However, the S169N mutation in isolation caused more complex unfolding/misfolding properties and it is important to remember that, like the double mutant MoPrP-N169N/N173T, this protein also has the ‘rigid loop’ phenotype^26^. Compared to the wildtype protein, we found that recMoPrP-S169N was less stable to both thermal- and urea-induced denaturation, but that it oligomerised more rapidly and fibrillised less rapidly than recMoPrP. It therefore behaved similarly to recMoPrP-S169N/N173T but was less stable to thermal denaturation.

Based on our studies (Table 1) and other published work^22^,^43^, we can offer the following interpretations: (i) Both of the non-‘rigid loop’ proteins that differed only by the N173T substitution produced similar results, both experimentally and *in silico*, hence the N173T mutation in isolation does not dramatically affect murine PrP^C^ structure or its misfolding pathways (ii) Both of the ‘rigid loop’ proteins that incorporated the S169N mutation fibrillised more slowly, oligomerised more rapidly and were less resistant to urea-induced denaturation than wildtype recMoPrP, indicating a key role for either the S169N substitution or the ‘rigid loop’ phenotype in driving unfolding/misfolding, however (iii) co-substitution of S169N and N173T produced a protein that was thermally more stable than recMoPrP suggesting that the ‘rigid loop’ phenotype *per se* does not determine overall protein stability. Instead, the available evidence suggests that the S169N substitution aids misfolding by helping to expose Tyr168 and that this occurs independently of the over-riding conformation/stability of the backbone of residues 166–168. Hence, although MoPrP-S169N/N173T has a ‘rigid loop’ phenotype and is thermally more stable than MoPrP, the degree of solvent exposure of Tyr168 also increases the oligomerisation kinetics of this protein. It is of considerable interest that the side chain of Tyr168 forms important interactions during steric zipper formation of a synthetic peptide from the β2-α2 region. Indeed, it has been shown that a hydrophobic amino acid at this location in PrP is a pre-requisite for efficient disease transmission^21^, whilst alteration of this amino acid to a range of other, non-hydrophobic residues reduces prion protein conversion^22^. In conclusion, our data do not support a role for the ‘rigid loop’ phenotype in mediating all unfolding/misfolding pathways of PrP, but suggest a complex interplay between the effects of specific amino acid substitutions that are unfolding/misfolding pathway-dependent.

## Experimental

### Cloning of recombinant prion protein variants

Cloning and expression of recombinant murine PrP (recMoPrP) spanning residues 23–230 has previously been described^44^. Mutated mouse prion protein constructs were prepared by site-directed mutagenesis (QuikChange II Kit from Stratagene). The double mutant (recMoPrP-S169N/N173T) was created by sequentially modifying both amino acids through two rounds of site directed mutagenesis. All expression plasmids were transformed into Rosetta 2 DE3 pLysS *E. coli* for protein expression and sequenced to confirm the presence of the appropriate nucleotide substitutions.

### Protein expression and purification

The method of expression of recombinant protein followed that previously published^27^. For thermal denaturation monitored by circular dichroism, the methods for protein purification follow those described previously^27^ and involved sequential purification by immobilised metal ion affinity chromatography (IMAC) followed by cation exchange chromatography under denaturing conditions. The final solution was dialysed extensively against 50 mM sodium acetate, pH 5.5, concentrated to ~1 mg/ml using centrifugal filtration and stored at −80 °C prior to use.

For fibrillisation and oligomerisation assays the purification method has also previously been described in detail^30^,^45^. The purification involves IMAC chromatography under reducing conditions followed by desalting on HiPrep 26/10 desalting column (GE Healthcare). The protein was further purified by HPLC using a reverse-phase column (214TP101522, Vydac), eluting the protein with a linear gradient from water to acetonitrile, acidified with 0.1% (v/v) trifluoroacetic acid. The protein was lyophilised in aliquots and stored at −20 °C prior to use.

For urea-based denaturation studies monitored by circular dichroism, protein was purified according to published protocols^32^. Solubilised inclusion bodies were separated initially on a Sephacryl S-300 gel filtration column (Amersham Biosciences, Buckinghamshire, U.K.), and recPrP was further purified by reversed-phase HPLC. Purified protein was dialyzed against 5 mM MES, pH 5 and stored in aliquots at −20 °C.

For all experiments, appropriate protein aliquots were thawed prior to use and their concentration determined spectrophotometrically using molar extinction coefficients calculated according to the method of Gill and von Hippel^46^.

### Thermal denaturation analysis by circular dichroism

The thermal stability assay of protein variants was performed and data processed as described previously^27^. Briefly, protein solutions were adjusted to 0.1 mg/ml and analysed by use of a Jasco J-710 spectropolarimeter. A sealed cuvette of 2 mm path length was heated from 20 °C to 90 °C. At each temperature point, 10 scans between 180–260 nm were taken and the reading at 222 nm was extracted. Multiple assays were performed for each protein variant, the data was normalised, averaged and fitted to a sigmoidal curve of the form x = M/(1 + r^(a–T)^), where ‘r’ is the rate of thermal denaturation in °C^−1^, ‘a’ is the midpoint of the transition, and ‘T’ is the temperature. ‘M’ represents the maximum unfolded percentage.

### Fibrillisation assay

Lyophilised recPrP was solubilised in 6 M guanidinium hydrochloride to a concentration of ~130 μM. The fibrillisation reaction was set up as described previously^29^,^30^. Briefly, recPrP stock was added to fibrillisation buffer where the final concentration in the reaction consisted of 2 M guanidinium HCl, 0.01 M thiourea, 0.05 M 2-(N-Morpholino)ethanesulphonic acid (MES) pH 6.0, 10 μM thioflavin T (ThT) and 5.2 μM recPrP. 160 μl of the reaction was added, per well, into a 96 well plate. For seeded fibrillisation assays, 1% (w/w protein) of homologous recPrP preformed into fibrils was added to the reaction mixture. Each well also contained three Teflon balls (3/32” diameter). The plate was constantly shaken at 900 rpm, 37 °C for 24 hours and ThT fluorescence was measured every 5 minutes, with excitation at 444 nm and emission at 485 nm. The increase in ThT fluorescence was plotted against time and a sigmoidal curve fitted to the data. The lag time of the sigmoidal curve was calculated as described previously^47^.

### Oligomerisation assay

The method to prepare oligomers from recPrP has been described before^27^,^31^. Lyophilised recPrP was solubilised in 20 mM sodium citrate, pH 3.4 buffer to a concentration of ~1 mg/ml and the protein was incubated at 45 °C for 2 hours. 15 μl aliquots of protein were analysed by size-exclusion chromatography using a TSKgelG4000SWxl gel filtration column (Tosoh Bioscience) with 20 mM sodium citrate as a running buffer at a flow rate of 1 ml/min. The column calibration and the identification of peaks has been described previously^27^. Within each experimental replicate, the percent of each species was plotted against reaction time and theoretical lines of best fit were calculated by use of Matlab, corresponding to the theoretical reaction described in Supplementary Materials. This process yielded multiple determinations of the kinetic constants for oligomerisation of each protein studied and significant differences between each were tested using two-tailed, student’s t-test assuming unequal variances. For the plots in Fig. 4(E–H), the percent intensity of each species was averaged and a line of best fit was calculated for each set of averaged data.

### Urea-based denaturation monitored by circular dichroism

Urea unfolding curves of recPrP variants were monitored by circular dichroism spectropolarimetry. For each experiment, folded and unfolded protein stocks were constructed in 8 M and 0 M urea respectively. At pH 7, samples were buffered with 20 mM sodium phosphate whilst at pH 4 the buffer was 20 mM sodium acetate. To prepare samples with intermediate amounts of urea, the correct proportions of unfolded and folded stock solutions were mixed.

The far-UV (190–260 nm) circular dichroism spectra were measured using JASCO J-815 spectropolarimeter by use of quartz cuvettes of path length 1 mm. A scanning rate of 100 nm/minute, time constant of one second, bandwidth of 1.0 nm, and resolution of 0.5 nm were used. Typically, 16 scans were averaged per spectrum and buffer backgrounds were subtracted from the final spectra. The mean residue ellipticity at 222 nm wavelength was determined for each CD spectrum recorded and normalised to the fraction unfolded, with ellipticity values at 8 M urea corresponding to fully unfolded state and those at 0 M urea to fully folded state.

### Molecular dynamics

The atomic coordinates representing the murine prion protein, residues 124–226, were downloaded from the protein data bank (PDB identification code 1AG2)^6^. This structure was used as a starting point for simulations by use of the AMBER suite of molecular dynamics software (version 7) using the Amber99 forcefield^48^. Coordinates for mutated prion proteins were calculated by use of Swiss PDB Viewer, in each case the energetically most stable enantiomer was chosen. All starting structures were extensively energy minimised to remove energy hot spots, structures were equilibrated *in silico* to 37 °C and molecular dynamics was carried out at 37 °C for 10 ns. A Born approximation of implicit solvent was used to expedite simulations whilst a simulation of 200 mM salt was included to maintain buffering. Coordinates were output every 10 ps during each simulation for analysis. Molecular trajectories were analysed using AMBER software or with VMD analysis software^49^ and figures were built using VMD or MOLMOL^50^.

## Additional Information

**How to cite this article**: Agarwal, S. *et al.* Complex folding and misfolding effects of deer-specific amino acid substitutions in the β2-α2 loop of murine prion protein. *Sci. Rep.* **5**, 15528; doi: 10.1038/srep15528 (2015).

## Supplementary Material

Supplementary Information

## Figures and Tables

**Table 1:** Summary of results from this work.

Protein	Rigid loop?	Fibril formation	Oligomer formation	Thermal denaturation	Urea-induced denaturation	Molecular dynamics
**recMoPrP**	No	Rapid	Slow	Medium	Stable	Loop region can adopt β-turn or 3–10 helix; Tyr168 can be either solvent exposed or protected
**recMoPrP-S169N**	Yes	Slow	Rapid	Less stable	Less stable	Reduced overall stability; Loop region can adopt β-turn or 3–10 helix; Tyr168 can be either solvent exposed or protected
**recMoPrP-N173T**	No	Rapid	Slow	Medium	Stable	Loop region can adopt β-turn or 3–10 helix; Tyr168 only solvent protected
**recMoPrP-S169N/N173T**	Yes	Slow	Very rapid and large oligomers formed	More stable	Less stable	Loop region only adopts a β-turn; Tyr168 only solvent exposed
