# Supplementary material for: Complex folding and misfolding effects of deer-specific amino acid substitutions in the β2-α2 loop of murine prion protein
Source: Sci Rep. 2015 Oct 22;5:15528. doi: 10.1038/srep15528 (PMC4614821; doi:10.1038/srep15528)
Supplement: Supplementary Information [file srep15528-s1.pdf]

Complex folding and misfolding effects of deer-specific amino acid substitutions in the β2-α2 loop of murine prion protein

Sonya Agarwal, Kristina Döring, Leszek Gierusz, Pooja Iyer, Fiona M. Lane, James F. Graham, Wilfred Goldmann, Teresa J. T. Pinheiro, Andrew C. Gill

Supplementary Data

Supplementary Figure 1

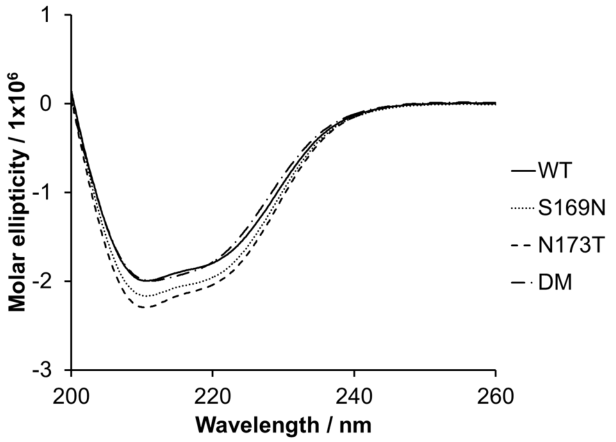

Far-UV circular dichroism spectra of normally-folded recombinant PrP: wildtype recMoPrP – solid line; recMoPrP-S169N – dotted line; recMoPrP-N173T – dashed line; recMoPrP-S169N/N173T – dot-dash line. All protein samples were adjusted to a concentration of approximately 1 mg/mL and a cell of path length 0.2 mm was used. Raw CD data were transformed to molar ellipticity for comparison. The units of molar ellipticity are deg cm^2/mol.

Supplementary Figure 2

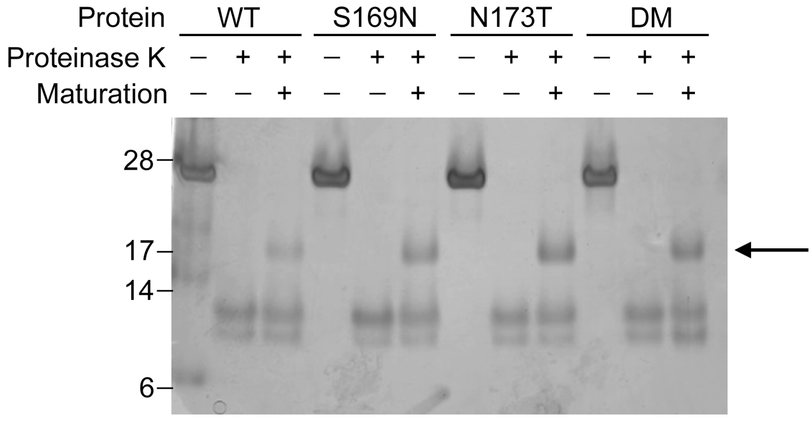

Proteinase K digestion of fibrils formed during fibrillization reaction. Formation of fibrils is confirmed by the presence of the 17 kDa band (arrowed) upon maturation of the fibrils [1] by heating at 80 °C for 15 minutes before addition of proteinase K. Proteins on the SDS-Page gel were visualised by silver staining.

1. Breydo, L., Makarava, N., and Baskakov, I.V. (2008). Methods for conversion of prion protein into amyloid fibrils. Methods Mol Biol *459*, 105-115.

Supplementary Figure 3:

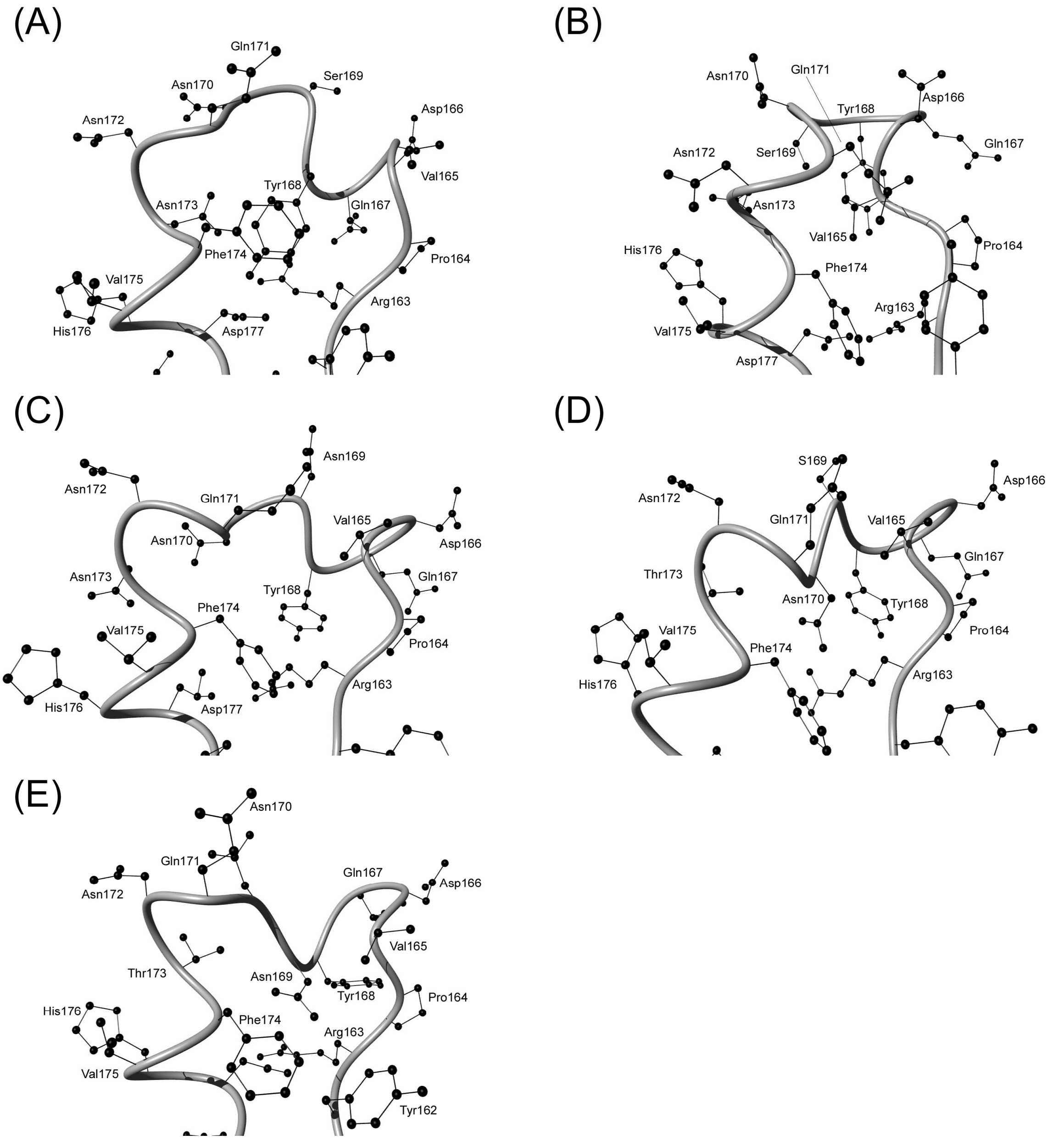

Schematic diagram of the β2-α2 loop region of the average structure calculated across the simulations of (A) MoPrP determined previously (B) MoPrP from this work (C) MoPrP-S169N (D) MoPrP-N173T (E) MoPrP-S169N/N173T. The backbone of each protein is shown as a continuous tube connecting the α-carbon atoms of each residue. The side chain of each amino acid is shown as a 'ball and stick' representation and is labelled. Schematics were produced in MolMol and rendered using PovRay.

Supplementary Figure 4

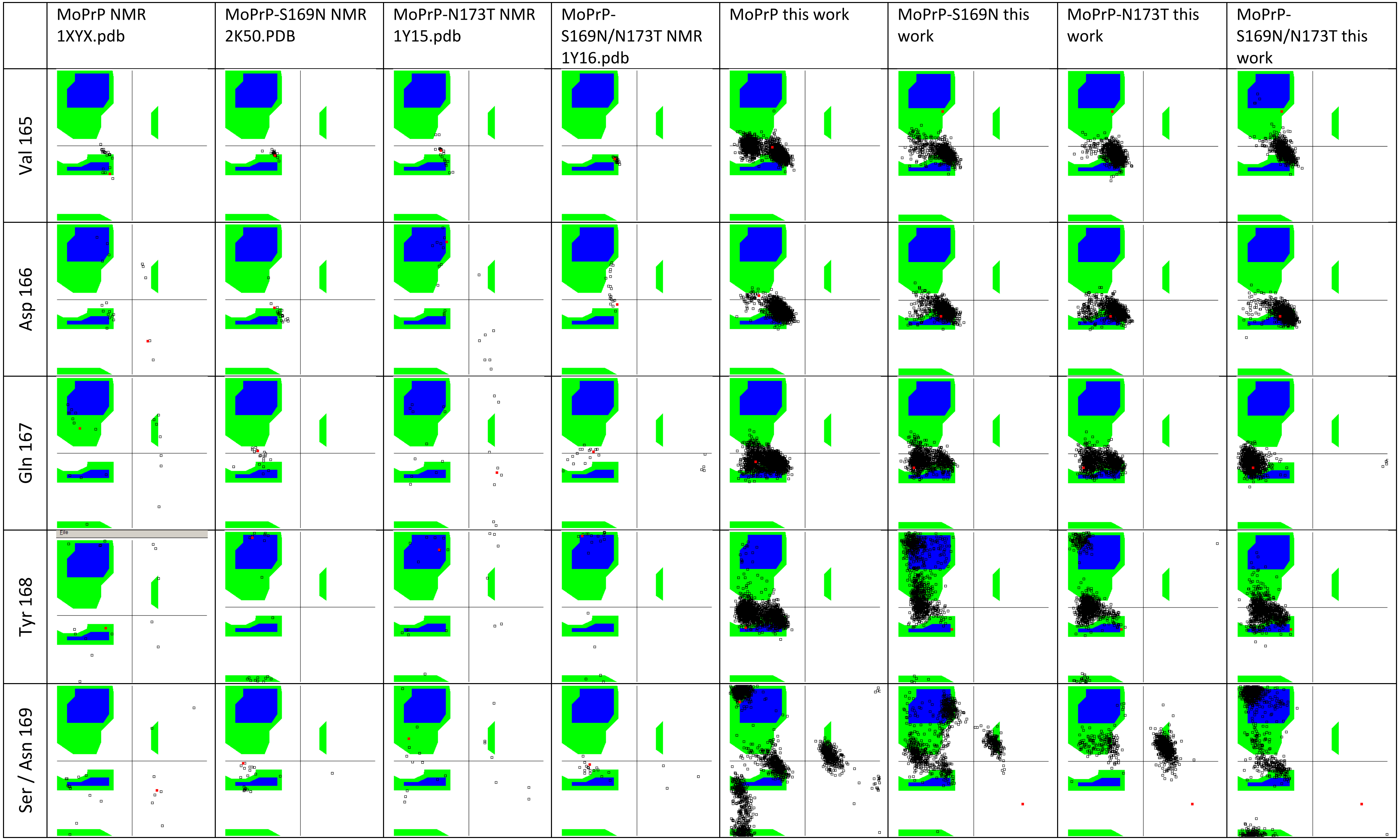

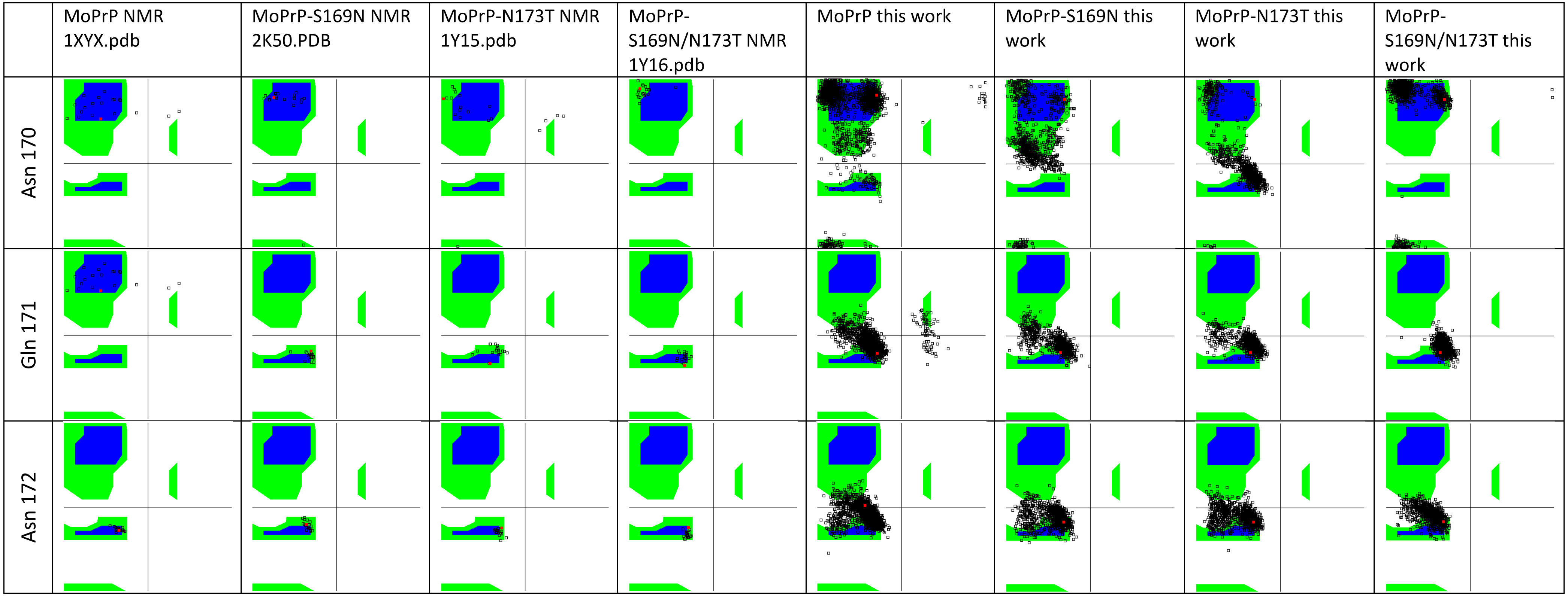

FIGURE 3: Ramachandran plots of backbone phi-psi angles in the β2-α2 loop region of the structures output from molecular dynamics simulations of the protein variants studied in this work. Also presented, for comparison, are the equivalent positions determined by from NMR spectroscopic analysis of the same recombinant proteins and published previously by Gossert [1] and Christen et al [2]. The coordinates for the NMR structures were obtained from the Protein Data Bank, as per the codes above and each NMR determined structural ensemble contains 20 possible structures. Ramachandran plots were generated by VMD [3].

1. Gossert, A.D., Bonjour, S., Lysek, D.A., Fiorito, F., and Wuthrich, K. (2005). Prion protein NMR structures of elk and of mouse/elk hybrids. Proc Natl Acad Sci U S A *102*, 646-650.
2. Christen, B., Perez, D.R., Hornemann, S., and Wuthrich, K. (2008). NMR Structure of the Bank Vole Prion Protein at 20 degrees C Contains a Structured Loop of Residues 165-171. J Mol Biol *383*, 306-312.
3. Humphrey, W., Dalke, A., and Schulten, K. (1996). VMD - visual molecular dynamics. Journal of Molecular Grpahics *14*, 33-38.

Supplementary Figure 5

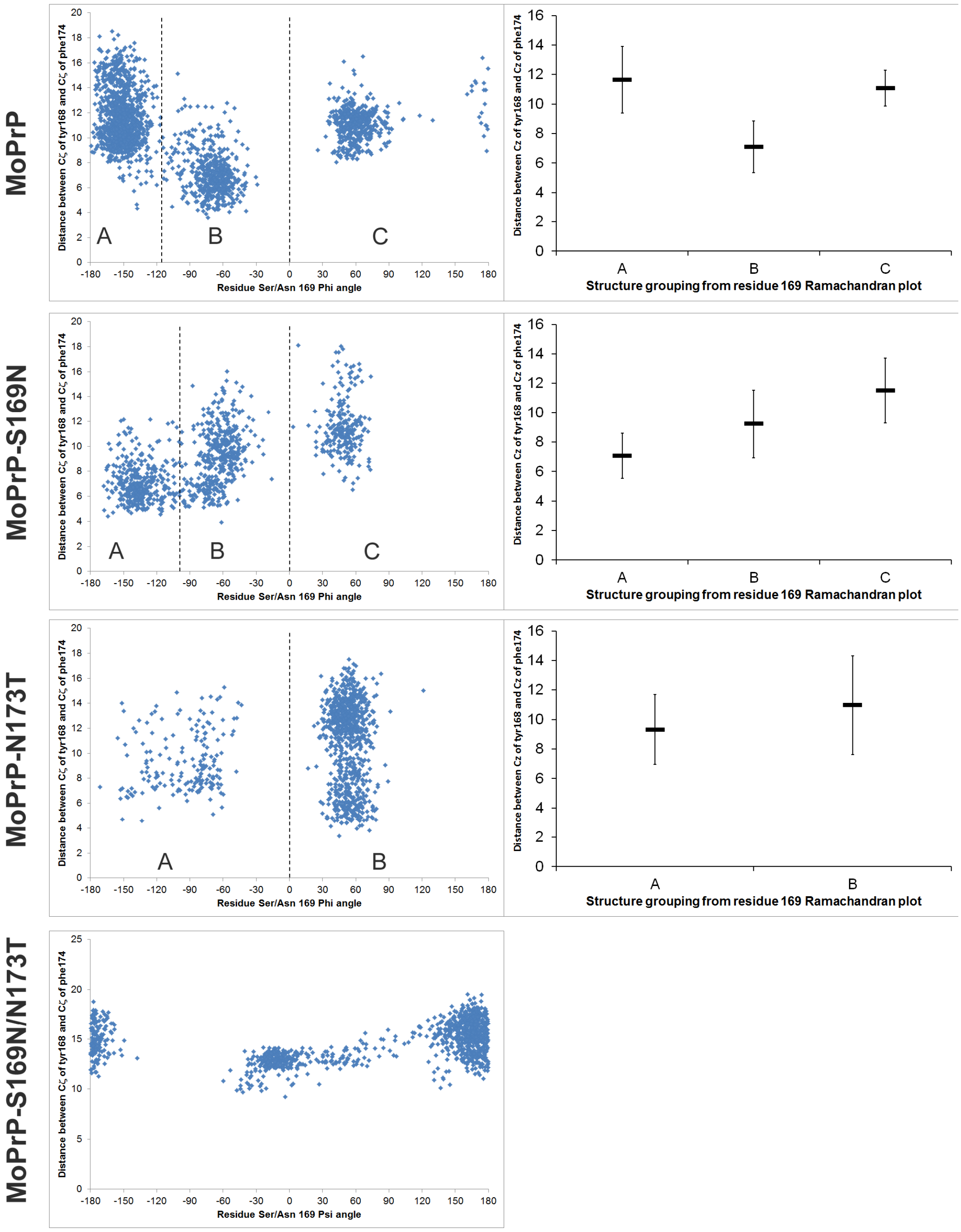

LEFT - Plots of the distance between the Cζ atom of tyrosine 168 and the Cζ atom of phenylalanine 174 as a function of the phi (or psi when this allows better separation) angle of residue 169 for each of the structures output during molecular dynamics simulations. For MoPrP, MoPrP-S169N and MoPrP-N173T proteins, obvious, discrete groups of protein structures resulted and the RIGHT graphs show the average Tyr168-Phe174 (Cζ atoms) separation for each of the groups. Error bars are the standard deviations.

Supplementary Table 1 Oligomerisation rate constants and statistical tests:

		k_1			
		**recMoPrP**	**recMoPrP-S169N**	**recMoPrP-N173T**	**recMoPrP-S169N/N173T**
k_2	**recMoPrP**		0.005	0.082	0.008
	**recMoPrP-S169N**	0.056		<0.001	0.423
	**recMoPrP-N173T**	0.063	0.356		0.002
	**recMoPrP-S169N/N173T**	0.082	0.138	0.108	

Two-way t-test statistics using two-tailed tests and assuming unequal variances.

Supplementary Experimental Procedures – oligomerization reaction kinetics

The oligomerization reaction has been investigated in detail by the group of Rezaie, but different putative reaction schemes have been published [1, 2]. These differ in terms of how the larger oligomer is formed; the initial report suggested that this species formed from the smaller oligomer, which in turn formed directly from the monomer. This yielded a 2 step, sequential, theoretical pathway. However, a later report suggested that formation of the oligomers represented competing pathways of oligomerization directly from the monomer; this suggestion produces a theoretical reaction pathway with parallel, competing, but reversible arms as depicted below and is more in line with our experimental data.

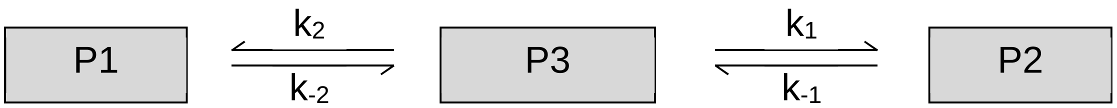

In this scheme, P3 represents the monomeric protein, P2 the small oligomer and P1 the large oligomer (nomenclature from Rezaie et al - ref). Thus, k1 is the rate constant of the reaction that generates the small oligomer from the monomer, whilst k-1 is the reverse reaction that reforms monomeric protein as the oligomer breaks down. It is assumed that both forward reactions are 3rd order (in terms of monomer) and that both reverse reactions are first order, as previously has been suggested by Rezaei. This reaction scheme yields the following differential, reaction rate equations which were fitted to the experimental data using Runge-Kutta ODE calculation procedures and least squares fitting in Matlab:

$$\frac{d[P3]}{dt} = k_{-1}[P2] + k_{-2}[P1] - 3k_1[P3]^3 + 3k_2[P3]^3$$

$$\frac{d[P2]}{dt} = 3k_1[P3]^3 - k_{-1}[P2]$$

$$\frac{d[P1]}{dt} = 3k_2[P3]^3 - k_{-2}[P1]$$

During the fitting procedure, the reverse rate constants k_{-1} and k_{-2} were each fixed at $1x10^{-5}$ s^{-1}. This value was chosen after many trials with different, fixed values for k_{-1} and k_{-2} were performed and $1x10^{-5}$ s^{-1} produced the best fits, as judged by the lowest sum of the squares of the errors.

1. Rezaei, H., et al., *Sequential generation of two structurally distinct ovine prion protein soluble oligomers displaying different biochemical reactivities.* J Mol Biol, 2005. **347**(3): p. 665-79.
2. Eghiaian, F., et al., *Diversity in prion protein oligomerization pathways results from domain expansion as revealed by hydrogen/deuterium exchange and disulfide linkage.* Proc Natl Acad Sci U S A, 2007. **104**(18): p. 7414-9.
